# Psychosocial and Physiological Factors Affecting Selection to Regional Age-Grade Rugby Union Squads: A Machine Learning Approach

**DOI:** 10.3390/sports10030035

**Published:** 2022-02-28

**Authors:** Julian Owen, Robin Owen, Jessica Hughes, Josh Leach, Dior Anderson, Eleri Jones

**Affiliations:** 1 Institute of Applied Human Physiology, School of Human and Behavioural Science, Bangor University, Bangor LL572DG, UK; j.owen@bangor.ac.uk (J.O.); jlhughes02@outlook.com (J.H.); 2 School of Health and Sport Sciences, Liverpool Hope University, Liverpool L169JD, UK; 3 Institute for the Psychology of Elite Performance, School of Human and Behavioural Science, Bangor University, Bangor LL572DG, UK; eleri.s.jones@bangor.ac.uk; 4 Welsh Rugby Union, Colwyn Bay LL297SP, UK; jleach@wru.wales; 5 Talent Pathway iD Ltd., Gaerwen LL606HE, UK; dior@talent-pathway.co.uk

**Keywords:** talent identification, talent selection, psychological factors, physical performance, pattern recognition, Bayesian machine learning, youth rugby

## Abstract

Talent selection programmes choose athletes for talent development pathways. Currently, the set of psychosocial variables that determine talent selection in youth Rugby Union are unknown, with the literature almost exclusively focusing on physiological variables. The purpose of this study was to use a novel machine learning approach to identify the physiological and psychosocial models that predict selection to a regional age-grade rugby union team. Age-grade club rugby players (*n* = 104; age, 15.47 ± 0.80; U16, *n* = 62; U18, *n* = 42) were assessed for physiological and psychosocial factors during regional talent selection days. Predictive models (selected vs. non-selected) were created for forwards, backs, and across all players using Bayesian machine learning. The generated physiological models correctly classified 67.55% of all players, 70.09% of forwards, and 62.50% of backs. Greater hand-grip strength, faster 10 m and 40 m sprint, and power were common features for selection. The generated psychosocial models correctly classified 62.26% of all players, 73.66% of forwards, and 60.42% of backs. Reduced burnout, reduced emotional exhaustion, and lower reduced sense of accomplishment, were common features for selection. Selection appears to be predominantly based on greater strength, speed, and power, as well as lower athlete burnout.

## 1. Introduction

Talent identification programmes assess the attributes of athletes, to guide talent selection programmes [1]. The aim of talent selection programmes is to select players with the potential to be the ‘sporting superstars’ of tomorrow and help clubs/governing-bodies achieve their long-term performance goals [2]. In furtherance of this long-term goal, selected players are usually integrated into talent development programmes which attempt to provide a learning environment that helps players achieve their potential [1]. However, talent selection programmes feature common problems. Firstly, youth performance is frequently used to predict success in adulthood when making selection decisions [3,4,5], despite youth performance offering low predictive accuracy [6,7,8]. For example, only 17% of male U18 sprinters who ranked among the top 50 highest performers internationally achieved the same ranking at senior level. Secondly, talent selection decisions are often made based on subjective criteria [1,3,4,9,10]. For example, interviews of national youth soccer coaches revealed that perceptions of talent and consequent selection decisions are primarily based on implicit coach preferences [9]. Consequently, current approaches to talent selection have been criticized [3,5] and more objective and evidence-based criteria are required to inform talent selection in sport [11,12,13,14]. 

Due to the physical nature of Rugby Union, players are generally required to have highly developed physical qualities [15]; players who are taller, heavier [16,17], faster [18], have greater strength [19], generate more power [20], and are relatively older compared to their peers [21], are more likely to be identified as having ‘talent’ and selected for development programmes. Within Rugby Union, there are also differences in the physical demands across positional units [22], especially within older age categories where position-specific fitness profiles are needed [23,24,25]. Notably, backs are involved in more high-intensity locomotor workload, whilst forwards perform more static high-intensity efforts than backs [26,27,28,29]. Accordingly, the physiological determinants of selection to talent development pathways appear to differ for forwards and backs; greater speed and agility has been shown to be an important talent selection criterion for backs, while greater upper-body strength, height, and mass are important talent selection criteria for forwards [16,30]. 

Although the physiological factors predicting selection to regional-age Rugby Union squads are relatively well understood, psychosocial factors (including personality) have received far less empirical attention [31]: with the exception being the work of Hill and colleagues [32,33]. It is generally accepted that selection/progression through elite performance pathways in other sports is facilitated by higher levels of emotional stability, coping strategies, perfectionism, optimism, extraversion, conscientiousness, emotional intelligence, agreeableness, discipline, self-confidence, resilience, and coachability [34,35,36,37,38,39,40]. Conversely, there is also evidence from other sports to suggest that certain psychosocial characteristics derail the development process of players [35]. For example, dysfunctional dispositions such as obsessive passion, maladaptive perfectionism, and dispositional optimism can negatively impact a player’s progression [41,42,43]. In accordance with findings from other sports, Hill et al. [33] reported that coaches perceived youth Rugby Union players as more likely to succeed if they exhibited greater proactiveness, commitment, growth-mindset, realistic performance evaluations, and resilience. It was reasoned that these skills help negotiate key challenges and developmental opportunities. They may also be a factor in reducing the likelihood of burnout brought forward by excessive perfectionism [32]. For example, greater resilience may enable individuals to persevere and stay engaged despite initial failures [33]. 

A limitation of Hill and colleagues’ [33] work is that conclusions were derived from retrospective coach opinions, rather than potentially more reliable player-based assessments. Additionally, their investigation primarily centred around progression through Rugby Union talent development programmes, forsaking psychosocial attributes’ role in Rugby Union talent selection. To address these gaps in the literature and satisfy recent calls to further investigate the role of psychosocial factors in Rugby Union talent selection [21], we utilized extensive primary physiological and psychosocial test batteries to differentiate between selected and non-selected regional age-grade Rugby Union players in North Wales (i.e., under 16 and 18 age categories). A novel Bayesian pattern recognition technique was used to identify which attributes (termed features in the analysis) differentiate between selection and non-selection. Thus, the present investigation offers an arguably more comprehensive test of factors than previous studies into age-grade selection [21]; is the first attempt to objectively understand the currently subjective decision-making that determines selection to regional age-grade academies in Wales; and tests the role of physiological attributes via new and cutting-edge analytical methods. Specifically, the Bayesian pattern recognition analysis we utilized accounts for the complex interaction between multiple variables when constructing models [44], provides a rigorous/conservative method to test the feature models that predict group classification (i.e., selected vs. non-selected) [45,46], and provides a way to explore interactions without potentially misleading assumptions/hypotheses [47]. Given the large number of physiological and psychosocial variables collected and relative exploratory nature of machine learning techniques, precise a priori hypotheses were not formed. However, it was anticipated that feature selection stages would identify similar predictive physiological variables to previous investigations in rugby union [31] and similar psychosocial variables to previous investigations in other sports [34,35,36,37,38,39,40].

## 2. Materials and Methods

### 2.1. Participants 

A total of 104 male U16 and U18 Rugby Union players (Mage = 15.47, SDage = 0.80; U16 *n* = 62; U18 *n* = 42) who attended one of two North Wales Rugby ‘Talent Camps’ in 2019 or 2020, volunteered to take part and gave informed consent in-line with institutional ethics guidelines. Of the 104 players who attended, 66 players were selected and 38 were not selected to the regional squads. Of the selected players, 37 were forwards (of which 16 = U16 and 21 = U18) and 29 were backs (of which 17 = U16 and 12 = U18). Of the non-selected players, 19 were forwards (of which 16 = U16 and 3 = U18) and 19 were backs (of which 13 = U16 and 6 = U18). These selections formed the six classification groups for analyses (i.e., selected players vs. non-selected players, selected forwards vs. non-selected forwards, and selected backs vs. non-selected backs). 

### 2.2. Procedure 

Players from regional squads and eligible age-grade clubs received an invitation to participate in a 1-day ‘talent camp’ in early spring 2019 or 2020, to assess their suitability for selection to a regional U16s or U18s rugby academy. Prior to these talent camps, players were advised to rest. During the talent camp, players completed a range of physiological and psychological assessments in a station-format which players rotated around until all tests were completed, followed by rugby matches. The selection decisions were made by regional coaches and based on subjective perceptions of performance during matches held on the talent days. For the purpose of this investigation, players were assessed on demographics, anthropometric, performance, and psychosocial measures (with the former 3 comprising 21 ‘physiological’ variables and the latter 47 ‘psychosocial’ variables) to identify differential features between those who were selected and not selected for the regional academy. 

Physiological demographic measures included self-reported weekly physical activity hours (assessed in 5-h increments, starting at 1–5 and going up to 30 h+), self-reported weekly training frequency with the academy before the talent camp (to the nearest integer), self-reported incidence of a significant injury during their career (assessed as ‘yes’ or ‘no’), and birth quarter (determined via birthday as: quartile 1 = September 1st to November 30th; quartile 2 = December 1st to February 28th/29th; quartile 3 = March 1st to May 31st; quartile 4 = June 1st to August 31st). For physiological anthropometric measures, players removed all heavy garments and footwear prior to recording measurements. Players’ body mass (kg) was measured using electronic column scales (Seca 799, GmbH, Hamburg, Germany). Standing height and sitting height (cm) were measured using a portable stadiometer (HR001, Tanita Europe BV, Amsterdam, The Netherlands) and leg length was calculated as the difference between standing and sitting height (cm). Body Mass Index (BMI) was calculated as weight divided by height (in metres) squared. The Reciprocal Ponderal Index (RPI), also known as Sheldon’s index [48], was calculated using the following equation: height (cm)/weight 0.333 (kg). Before measurement of physical performance measures, all participants completed a standardised (in terms of time and intensity) warm-up administered by regional strength and conditioning coaches and were briefed on how to execute each assessment. The counter movement jump was performed on a jump mat (JustJump, Probiotics Inc, Huntsville, AL, USA) indoors while wearing trainers, to assess jump height (cm) and peak anaerobic lower body power (W) using the Sayers Equation [49]; hands were positioned on the hips and the best jump height from three trials was recorded [50]. A hand grip strength test (Takei 5001 Grip-A Handgrip Dynamometer, Takei Scientific Instruments Co, Nigata, Japan) was used to infer strength (kg) within the dominant and non-dominant arm; participants stood with their back against a wall with their testing arm at 10°–15° from the shoulder and elbow flexed at 90° with the highest score from two attempts (per arm) recorded [51]. Time (s) taken to sprint 10 and 40 m was recorded using timing gates (Brower Timing Systems, Draper, USA) on a 3G artificial grass pitch while wearing rugby shoes with studs; each sprint distance was completed twice with a 2-min rest between each repetition, with the fastest time recorded for each player. For the 40 m sprint: velocity was calculated as 40 m divided by the time taken to complete the 40 m; acceleration was calculated as velocity divided by the time taken to complete the 40 m sprint; force was calculated as acceleration multiplied by weight (kg); momentum was calculated as the velocity multiplied by the player’s weight (kg); and average power was calculated using the Harman Formula [52]. 

The psychosocial questionnaires were administered in two questionnaires’ packs to players during the 1-day ‘talent camp’. Players were informed that their responses would not affect their selection. The first questionnaire pack gathered training behaviours (e.g., goal orientation, commitment, athlete identity). Players were also asked to report the number of hours of employed work they completed every week. The second questionnaire pack examined competitive experiences and personality traits (e.g., optimism, perfectionism, alexithymia). Questionnaires were chosen based on previous research which has identified these psychological constructs as important for athlete development [38]. In order to include several components and to circumnavigate issues with excessive questionnaire length, two items per construct were included. For complete information on the psychosocial variables collected, original sources, and items used, see Appendix A. 

### 2.3. Data Analysis 

To evaluate which features (i.e., predictor variables) best classified (i.e., determined) group membership (selected vs. non-selected), Bayesian pattern recognition was performed; a complete list of the features evaluated by the pattern recognition analysis (21 physiological and 47 psychosocial variables) can be found in Table 1. To explore the relative importance of factors within their topic-domains and player-positional categories and reduce the likelihood of machine learning overly reducing the features considered within datasets, we split analyses by positions (i.e., backs and forwards) and domains (i.e., physiological and psychosocial). Pattern recognition was performed using the open-source programming language R (R Core Development, 2021). Within this coding environment, the Tidyverse package [53] was used to perform advanced data manipulation, and the rWeka package [54]) was used to interface R with WEKA machine learning algorithms [55]. Analysis comprised three stages: first, features were standardised as part of data pre-processing; second, feature selection was performed to filter the dataset to a combination of its most predictive features, thus creating ‘models’ of features that best at differentiated group classification; and third, the classification accuracy of the created models was tested to evaluate how well the created models should predict group membership in future. 

### 2.4. Pre-Processing 

For all analyses, the data of U16 and U18 players were standardised and amalgamated. The raw U16 data were transformed into z-scores using the U16 means and standard deviations, and the raw U18 data was transformed into z-scores using the U18 means and standard deviations. Therefore, when the z-scored U16 and U18 data were amalgamated, z-scores indicated how much greater/less athletes scored on a feature (i.e., predictor variable) compared to their age-group peers. For data processing purposes, each z-scored feature was converted into a vector that went from 0 to 100 (with a player’s score of 50 representing a score equivalent to the age-group mean and a score of 60 represented 1SD above the age-group mean, etc.). The purpose of amalgamating the data of U16 and U18 players was to: construct/evaluate classification models with greater accuracy via a larger dataset; identify features which determine overall ‘age-grade’ rugby union selection; and aid interpretation because similar features and model classification accuracies emerged for U16 and U18 players when analysed separately.

### 2.5. Feature Selection for Model Creation 

Feature selection involved the use of correlation attribute evaluator [56], relief F attribute evaluator [57], gain ratio attribute evaluator [58], and info gain attribute evaluator [58], to identify (up to) 15 of the strongest features for determining group membership (i.e., selected vs. non-selected). Only features which were identified as being in the top 15 (this criterion was set arbitrarily based on the number of variables collected, prior to any data analysis) by at least two feature selection algorithms could become part of a ‘model’ and proceed in the analysis (some of the feature selection algorithms used can return less than 15 features if they are deemed as insufficiently predictive [56,57,58]). The resulting models were the combination of features within the dataset that best predicted group classification. Feature selection was performed a total of 6 times to create 6 models for 3 position conditions (all players, forwards, and backs) × 2 feature subsets (physiology features and psychosocial features). 

### 2.6. Model Classification Accuracy 

Each of the six models created by feature selection stage had its classification accuracy tested (i.e., how accurate, in percentage terms, a model is in predicting group membership) via the use of Naïve Bayes [59], J48 decision tree [60], Support Vector Machine [61], and K-nearest neighbour [62] classification algorithms. These algorithms assigned each player with an expected group membership (selected or non-selected) based on their score on features within the model. This process was iterated using a ‘leave one out’ cross-validation procedure wherein classification algorithms were performed repeatedly but with each of players’ data left out once [45]. Thus, the final classification accuracy reported was the average percentage accuracy across each iteration. This ‘leave one out’ cross validation procedure was chosen over a training/validation sample-split to create the most accurate-possible models (i.e., via an as-large-as-possible dataset during feature selection), whilst still minimizing the overfitting of results to the specific dataset and preserving generalizability (i.e., via the conservative nature of the ‘leave one out’ method) [45].

## 3. Results

Table 2 contains the models created by feature selection and their overall classification accuracy. All models comprised between three and six features; naturally, less features were agreed on by more feature selection algorithms. Classification accuracy of the models ranged between 60 and 72% and was better than chance. 

Table 3 displays the classification analysis of the physiological feature model created from all players. Overall, this model was able to correctly classify players 67.55% of the time. Specifically, selected players were faster in 10 m sprint (1.81 ± 0.13 vs. 1.83 ± 0.10 s), had greater power over 40 m (748.96 ± 131.91 vs. 600.72 ± 137.88 W), greater dominant (43.52 ± 6.54 vs. 39.78 ± 7.96 kg) and non-dominant hand grip strength (40.23 ± 7.07 vs. 36.97 ± 7.80 kg) than non-selected players.

Table 4 displays the classification analysis of the psychosocial feature model created from all players. Overall, this model was able to correctly classify players 62.26% of the time. Specifically, selected players had lower reduced sense of accomplishment (10.20 ± 2.61 vs. 11.08 ± 2.19 questionnaire score), lower burnout (27.12 ± 5.79 vs. 29.37 ± 5.99 questionnaire score), lower exhaustion (9.15 ± 2.69 vs. 10.24 ± 3.55 questionnaire score), and lower introjected regulation’ (4.12 ± 2.53 vs. 4.82 ± 3.14 questionnaire score) than non-selected players. 

Table 5 displays the classification analysis of the physiological feature model created from forwards. Overall, this model was able to correctly classify players 70.09% of the time. Specifically, compared to non-selected players, selected players had faster 10 m (1.85 ± 0.14 vs. 1.86 ± 0.10 s) and 40 m sprint times (5.74 ± 0.32 vs. 5.90 ± 0.36 s), expressed greater force (106.96 ± 14.65 vs. 83.60 ± 10.82 N) and power over 40 m sprints (747.92 ± 112.56 vs. 570.04 ± 89.89 W), had lower reciprocal ponderal index (40.63 ± 2.03 vs. 41.78 ± 1.79 cm kg 0.333) and greater non-dominant hand grip strength (40.18 ± 7.24 vs. 36.26 ± 7.57 kg). 

Table 6 displays the classification analysis of the psychosocial feature model created from forwards. Overall, this model was able to correctly classify players 73.66% of the time. Specifically, selected players had lower life stress (6.97 ± 2.22 vs. 8.89 ± 2.85 questionnaire score), lower reduced sense of accomplishment (10.05 ± 2.73 vs. 11.21 ± 2.74 questionnaire score) and lower difficulty describing feelings (3.84 ± 1.64 vs. 5.05 ± 1.93 questionnaire score) than non-selected players. 

Table 7 displays the classification analysis of the physiological feature model created from backs. Overall, this model was able to correctly classify players 62.5% of the time. Specifically, selected players had faster 40 m sprint times (5.40 ± 0.26 vs. 5.61 ± 0.41 s), greater momentum in the 40 m sprints (540.40 ± 76.31 vs. 481.84 ± 82.26 kg·m·s^−1^), were born in an earlier birth quarter (2.10 ± 1.01 vs. 2.68 ± 1.20) and had greater dominant hand grip strength (43.05 ± 5.99 vs. 40.84 ± 9.13 kg) than non-selected players.

Table 8 displays the classification analysis of the psychosocial feature model created from backs. Overall, this model was able to correctly classify players 60.42% of the time. Specifically, selected players had lower introjected regulation (4.10 ± 2.60 vs. 5.26 ± 3.19 questionnaire score), lower reduced sense of accomplishment (10.38 ± 2.50 vs. 10.95 ± 1.51 questionnaire score) and lower burnout (27.93 ± 5.66 vs. 30.16 ± 6.69 questionnaire score) than non-selected players.

## 4. Discussion

The aim of this study was to measure primary physiological and psychosocial factors in age-grade Rugby Union players and to utilize a novel Bayesian pattern recognition technique to identify which attributes differentiate between selection and non-selection to regional U16 and U18 performance pathways. The main findings of this investigation suggested that the generated physiological models correctly classified 67.55% of all players, 70.09% of forwards, and 62.50% of backs. Greater hand-grip strength, faster 10 m and 40 m sprint, and power were common features for selection. The generated psychosocial models correctly classified 62.26% of all players, 73.66% of forwards, and 60.42% of backs. Reduced burnout and emotional exhaustion, and lower reduced sense of accomplishment, were common features for selection. Selection appears to be predominantly based on greater strength, speed, and power, as well as lower athlete burnout. Of note, the greater specificity and lower sensitivity across all analyses suggests that non-selected players were easier for the algorithms to identify. This finding is logical when one considers that players who should not be selected likely stand out more (e.g., particularly slow/weak) compared to players who should be selected (i.e., where the margins may be finer). The present investigation offers an arguably more comprehensive test of factors than previous studies into age-grade rugby selection (e.g., [21]); is the first attempt to objectively understand the currently subjective decision-making that determines selection to regional age-grade academies in Wales; and tests the role of psychosocial and physiological attributes via new and cutting-edge analytical methods.

The findings of this investigation provide a unique insight into differences in psychosocial components between selected and non-selected players. The results suggest that selected players (generally across positions) reported lower levels of overall burnout and specifically lower exhaustion and lower reduced sense of accomplishment compared to non-selected players. Consistent with previous research in Rugby Union [32,33], these results suggest that burnout is a prominent factor in the sport. Interestingly, the present pattern recognition analysis did not support previously proposed theoretical explanations of burnout, exhaustion, and reduced sense of accomplishment, such as perfectionism and coping [32,63]; it is possible that the mechanisms producing these outcomes were too individualized within the present sample to be identified at the feature selection stage. Regardless of the precise mechanisms leading to burnout, results highlight the need for coaches to consider how it could ultimately derail athlete progression within talent selection and development [10].

The psychosocial results also reveal differences across forwards and backs. Forwards report lower life stress, which is logical when viewed in line with results on burnout [64]. The selected forwards also reported lower scores in difficulties describing feelings, which is a component of the personality trait Alexithymia [65]. Those high in Alexithymia often are unable to express and recognise their emotions leading to difficulties in regulating emotions and difficulties with interpersonal relations [65]. Alexithymia is relatively under-researched in athletes, but some research has linked those high in Alexithymia with risk-taking [66], and endurance sports [67]. From a forward’s perspective, it could be argued that the lower scores related to difficulty describing emotions are indicative of greater emotional regulation, and their ability to resolve negative emotions that arose from stressful aspects of life [67] and physical demands of playing forward. This is again logical when considered with the reports of lower life stress and burnout. Future research should attempt to tease the present findings apart by further investigating the coping strategies that might differ across positions. 

Whilst research has shown positional differences from a relative age effect [68] and physiological perspective [69], the present investigation is, to the authors’ knowledge, the first to reveal positional differences in psychosocial components of selected vs. non-selected rugby players. Research by Dimundo et al. [21] included one measure of cognitive skills in rugby union players but found no significant differences across players and went on to call for future research to include psychosocial characteristics in talent identification/selection methodologies. Adopting a battery of psychological tests comprising fewer items in the present investigation provided an opportunity to assess players on a wide variety of relevant psychosocial components. Recent applied research [70,71] has also adopted this method of utilizing fewer items per construct to facilitate both a broad assessment and to encourage athlete engagement. Whilst we would recommend that any psychosocial investigations such as this are followed up on a more detailed basis between a sport psychologist and coaches, the method adopted here does facilitate a broad understanding of psychosocial component relevant to talent identification, selection, and development. 

Physiological models correctly classified selected players in the range between 62.50% and 70.09% and were stronger predictors of selection than psychosocial models, which has been alluded to previously [21]. In addition, the common features for selection within our models are generally in agreement with previous research examining differences in physical and performance measures between selected and non-selected players. For example, in the present investigation, greater hand grip strength was a performance feature important for selection across all players and within positional categories. Others have confirmed that greater strength in general [72,73] and handgrip strength specifically [19,21,74] is a characteristic of selection to rugby performance pathways and distinguishes between standard of play in age-grade players [75]. 

Sprinting speed is an important physical quality in Rugby Union and is associated with many performance parameters such as evading opponents, line and tackle breaks and has been shown to distinguish between selected and non-selected age-grade players [18,31,73,75]. In the present investigation, selected players recorded faster sprint times over 10 m (all players and forwards) and 40 m (forwards and backs). Indeed, 10 m sprinting speed was one of the features within the model that correctly classified 67.55% of all players, and coaches and sport scientists should ensure the inclusion of these assessments into talent selection programmes. 

Previous research has consistently shown that selection for Rugby Union performance pathways across U15–U21 age grades is biased towards taller [73,75] and heavier players [21,75]. This may explain the well-established selection bias towards relatively older players [31,76,77,78] and to some degree early maturing players [79] (i.e., the relative age effect). Notably, in the present investigation, stature and body mass were not common features of selection for all players regardless of positional category. Although earlier birth quartile (Q1 and Q2) was part of the model for selected backs, this was not a feature for selection in the physiological models covering all players and forwards and may partly explain these findings. Despite its absence as a direct feature for selection, body weight did appear to be an important factor when expressed as momentum (backs), force (forwards) and power during 40 m sprinting (all players and forwards). Further suggestion of the importance of body shape and size was evidenced via a lower Reciprocal Ponderal Index (RPI) as an important feature for selection for forward players. The RPI is an index of adiposity calculated as the relationship between standing height divided by the cube root of body weight and based on allometric modelling has a stronger mathematical foundation than BMI, as weight is a variable of cubic dimensions [48]. RPI has been associated with performance in sports such as soccer and tennis [80,81]. The lower RPI found in selected forwards in the present investigation would infer that greater body mass rather than a more linear (ectomorphic) body shape is an important factor in terms of selection within this positional category. 

The methods used to derive the aforementioned psychosocial and physiological findings feature several strengths. Firstly, the present investigation was the first to directly assess the role of primary player-derived psychosocial attributes on talent selection in Rugby Union. Secondly, the novel pattern recognition analysis performed on physiological features revealed similar predictive features for talent selection in Rugby Union to previous correlational studies [31] which, importantly, gives confidence to the psychosocial features identified as predictive for the first time. Lastly, a rigorous and conservative ‘leave one out’ cross-validation classification procedure was used. This classification procedure facilitates more accurate feature models (i.e., via an as-large-as-possible dataset during feature selection) whilst minimizing the overfitting of results to the specific dataset (i.e., by testing classification accuracy on the entire sample, instead of a small validation-specific sample) [45,46]. The newfound knowledge from the present investigation can be used by coaches, managers, parents, and guardians in making sure youth Rugby Union players are adequately developed and supported for future success. Coaches may wish to prioritize the physiological development of relatively stronger and faster players, while parents and guardians may wish to monitor for signs and causes related to burnout and exhaustion. Such provision should position Rugby Union players optimally for selection by regional age-grade academies. 

It is important to note however, some of the present investigation’s limitations. Classification accuracies (60–74%) were less than those of studies utilizing similar machine learning approaches in other sporting domains [82,83]. However, this result can be expected for two reasons. The regional academy’s subjective/intuition-based selection criteria likely introduce inevitable statistical ‘noise’, and the present investigation’s conservative ‘leave one out’ cross-validation classification procedure likely resulted in lower classification accuracies. One method to increase classification accuracy despite this, could be the use of even more comprehensive test batteries (e.g., via evaluations of practice histories, technical ability, tactical ability, and performance history). For example, evidence to suggest that the features collected in our study do not capture the role of tactical/technical attributes in determining selection, can be seen in the backs’ generally lower classification accuracy (~60%) compared to forwards’ (~70). For backs in particular, tactical and technical skill may be a particularly important trait when academies evaluate players. Future studies are encouraged to collect ratings of players’ tactical and technical ability from independent coaches, alongside developmental variables such as practice histories, which have demonstrated themselves as important factors in determining future success [83]. Additionally, subsequent investigations may wish to also evaluate the interactive role of aerobic fitness, a variable that was not possible to assess in the present investigation due to time constraints on the talent camp day but has previously demonstrated an ability to differentiate between selected and non-selected rugby union players [31]. 

## 5. Conclusions

This is the first study that has utilized a machine learning approach to examine the factors that determine selection to a regional age-grade Rugby Union academy in Wales. The present investigation offers an arguably more comprehensive analysis of factors than previous studies in this population and informs an objective understanding of the current subjective decision-making that determines selection to regional age-grade academies in Wales. From these findings, it appears that physiological factors are more predictive of selection. Specifically, the findings of this present investigation suggest that greater strength, speed, and power during sprint running were important factors for selection and should be included as routine assessments in talent selection for regional academies. Nevertheless, psychosocial factors were also shown to be important with reduced burnout and emotional exhaustion, and lower reduced sense of accomplishment, common features for selection. Indeed, this is the first study to comprehensively examine psychosocial factors important for selection to rugby academies and the findings add weight to the argument that these factors should be considered as part of a holistic selection framework in Rugby Union. Furthermore, practitioners should also consider position-specific differences in factors important for selection when planning talent selection frameworks. Future studies are encouraged to adopt a holistic approach to talent selection through investigating a comprehensive combination of physiological and psychosocial factors alongside tactical and technical ratings and developmental variables such as practice histories.

## Figures and Tables

**Table 1:** Pattern recognition variable list.

Physiological Variables				
Weekly Physical Activity Hours	Weekly Academy Training Frequency	Injury Occurrence During Career	Birth Quarter	Height
Weight	Sitting Height	Leg Length	Reciprocal Ponderal Index	BMI
Counter Movement Jump	Dominant Hand Grip Strength	Non-Dominant Hand Grip Strength	10 m Sprint Time	40 m Sprint Time
40 m Sprint Momentum	40 m Sprint Velocity	40 m Sprint Acceleration	40 m Sprint Force	40 m Sprint Power
Peak Anaerobic Lower Body Power				
**Psychosocial Variables**				
Weekly Hours of Employed Work	Goal Orientation	Outcome Focus	Mastery Focus	Commitment to Training
Burnout	Exhaustion	Reduced Sense of Accomplishment	Sport Devaluation	Life Stress
Training Stress	Athlete Identity	Optimism	Difficulty Describing Feelings	Difficulty Identifying Feelings
Externally Orientated Feelings	Perfectionism	Perfectionistic Concerns	Perfectionistic Strivings	Self-Esteem
Extraversion	Agreeableness	Conscientiousness	Emotional Stability	Openness to New Experiences
Motivation	Amotivation	External Regulation	Introjected Regulation	Identified Regulation
Integrated Regulation	Intrinsic Motivation General	Resilience	Emotional Intelligence	Appraisal of own Emotions
Appraisal of Others’ Emotions	Regulation of own Emotions	Regulation of Others’ Emotions	Utilisation of Emotions	Coping Strategies
Coping with Adversity	Peaking under Pressure	Goal Setting and Mental Preparation	Concentration	Freedom from Worry
Confidence and Achievement Motivation	Coachability			

Note. A total of 21 physiological variables (comprising demographic, anthropometric, and performance measures) and 47 psychosocial variables were entered into the feature selection stage.

**Table 2:** Algorithm agreement among feature selection models and classification.

	Models					
	All Players		Forwards		Backs	
**Number of feature selection algorithms in agreement**	Physiological Features	Psychosocial Features	Physiological Features	Psychosocial Features	Physiological Features	Psychosocial Features
**4**		- Reduced sense of accomplishment.		- Life stress.		
**3**	- Power over 40 m.		- Force over 40 m; - Power over 40 m.		- Momentum;	
**2**	- 10 m sprint; - 40 m sprint; - Dominant hand grip strength; - Non-dominant hand grip strength.	- Burnout; - Exhaustion; - Introjected regulation.	- 40 m sprint; - Reciprocal Ponderal Index - Non-dominant hand grip strength; - 10 m sprint.	- Reduced sense of accomplishment; - Difficulty describing feelings;	- Birth quarter; - 40 m sprint; - Leg length; - Dominant hand grip strength.	- Reduced sense of accomplishment; - Introjected regulation; - Burnout
Classification accuracy	67.55%	62.26%	70.09%	73.66%	62.5%	60.42%

Note. The columns in this table contain the features within each group’s model from feature selection. A greater number of algorithms agreeing that a feature is of great predictive validity, gives increased confidence in this feature belonging in the model. Classification accuracy is the mean accuracy (in percent) across all model levels (2, 3, and 4) and classification algorithms.

**Table 3:** Classification breakdown of all players’ physiological model.

Classifier	Classification Accuracy (%)	Sensitivity	Specificity	Area under ROC Curve
Naïve Bayes	67.31	0.45	0.80	0.63
Support Vector Machine	63.46	0	1	0.5
K Nearest Neighbour	69.23	0.45	0.83	0.68
J48 Decision Tree	70.19	0.34	0.91	0.43
Mean	67.55	0.31	0.89	0.56

Note. Sensitivity = 1—false positive. Specificity = 1—false negative. ROC = Receiver operating characteristic.

**Table 4:** Classification breakdown of all players’ psychosocial model.

Classifier	Classification Accuracy (%)	Sensitivity	Specificity	Area under ROC Curve
Naïve Bayes	64.42	0.37	0.80	0.61
Support Vector Machine	63.46	0	1	0.5
K Nearest Neighbour	59.61	0.24	0.80	0.54
J48 Decision Tree	61.53	0.50	0.68	0.57
Mean	62.26	0.28	0.82	0.56

Note. Sensitivity = 1—false positive. Specificity = 1—false negative. ROC = Receiver operating characteristic.

**Table 5:** Classification breakdown of the forwards’ physiological model.

Classifier	Classification Accuracy (%)	Sensitivity	Specificity	Area under ROC Curve
Naïve Bayes	69.64	0.53	0.78	0.74
Support Vector Machine	76.79	0.53	0.89	0.71
K Nearest Neighbour	73.21	0.53	0.84	0.73
J48 Decision Tree	60.71	0.58	0.62	0.52
Mean	70.09	0.54	0.78	0.68

Note. Sensitivity = 1—false positive. Specificity = 1—false negative. ROC = Receiver operating characteristic.

**Table 6:** Classification breakdown of the forwards’ psychosocial model.

Classifier	Classification Accuracy (%)	Sensitivity	Specificity	Area under ROC Curve
Naïve Bayes	75.00	0.53	0.86	0.67
Support Vector Machine	62.29	0	0.97	0.49
K Nearest Neighbour	73.21	0.47	0.86	0.65
J48 Decision Tree	82.14	0.53	0.97	0.51
Mean	73.16	0.38	0.92	0.58

Note. Sensitivity = 1—false positive. Specificity = 1—false negative. ROC = Receiver operating characteristic.

**Table 7:** Classification breakdown of the backs’ physiological model.

Classifier	Classification Accuracy (%)	Sensitivity	Specificity	Area under ROC Curve
Naïve Bayes	68.75	0.47	0.83	0.61
Support Vector Machine	54.17	0	0.90	0.45
K Nearest Neighbour	58.33	0.26	0.79	0.59
J48 Decision Tree	68.75	0.26	0.97	0.26
Mean	62.50	0.24	0.87	0.48

Note. Sensitivity = 1—false positive. Specificity = 1—false negative. ROC = Receiver operating characteristic.

**Table 8:** Classification breakdown of the backs’ psychosocial model.

Classifier	Classification Accuracy (%)	Sensitivity	Specificity	Area under ROC Curve
Naïve Bayes	62.50	0.26	0.86	0.63
Support Vector Machine	58.33	0	0.97	0.48
K Nearest Neighbour	66.67	0.37	0.86	0.69
J48 Decision Tree	54.17	0	0.90	0
Mean	60.42	0.16	0.90	0.45

Note. Sensitivity = 1—false positive. Specificity = 1—false negative. ROC = Receiver operating characteristic.

## Data Availability

Data can be obtained from the authors.

## Appendix A

**Table A1:** Summary of measures used in psychological questionnaire packs 1 and 2.

Measure and Item Origin	Subscale	Items from Original Construct	Factor Loading	Author
TRAINING BEHAVIOURS				
Perception of Success (Roberts, Treasure, and Balague, 1998)	Outcome Focus	1. When doing sport, I feel successful when I beat other people.	0.66	Items taken from the ADFS (Dunn et al., 2019)
		2. When doing sport, I feel successful when I outperform my opponents.	0.62	
	Mastery Focus	1. When doing sport, I feel successful when I perform to the best of my ability.	0.62	
		2. When doing sport, I feel successful when I show clear personal improvements.	0.72	
Quality of Training Inventory (Woodman et al., 2010)	Commitment to Training	1. I always produce a high-quality training session.		Items taken from the ADFS (Dunn et al., 2019)
		2. No matter what is going on in my life, I still turn in a good training session.		
Inclusion of Others in the Self Scale (Aron, Aron, and Smollan, 1992)	Athlete Identity	1. My sport is the most important thing in my life.		Items taken from the ADFS (Dunn et al., 2019)
		2. My sport offers me more than anything else in life (e.g., friends, family, relationships, money).		
Behavioural Regulation in Sport (Lonsdale, Hodge, and Rose, 2008)	Amotivation	1. but I question why I continue.	0.90	Items taken from the BRSQ-6 (Lonsdale, Hodge, and Rose, 2008)
		2. but the reason why are not clear to me anymore	0.89	
	External Regulation	1. because people push me to play	0.85	
		2. because I feel pressure from other people to play	0.84	
	Introjected Regulation	1. because I would feel guilty of I quit	0.78	
		2. because I fee; obligated to continue	0.88	
	Identified Regulation	1. because the benefits of sport are important to me	0.80	
		2. because it teaches me self-discipline	0.57	
	Integrated Regulation	1. because it’s an opportunity to just be who I am	0.70	
		2. because what I do in sport is an expression of who I am	0.77	
	IM-General	1 because I enjoy it	0.82	
		2. because I like it	0.81	
Performance-based Self-Esteem (Hallsten, Josephson, and Torgén, 2005)	Self-Esteem	1. I think that I can sometimes try to prove my worth by being competent.	Range from 0.70 to 0.84	Items taken from the Pbse-scale (Hallsten, Josephson, and Torgén, 2005)
		2. My self-esteem, is far too dependent on my daily achievements.		
		3. At times, I have to be better than others to be good enough myself.		
		4. Occasionally I feel obsessed to accomplish something of value.		
Athlete Coping Skills Inventory-28 (Smith, et al., 1995)	Coping with Adversity	1. I maintain emotional control no matter how things are going for me.	0.60	Items taken from the ACSI-28 (Smith, et al., 1995)
		2. When things are going badly, I tell myself to keep calm, and this works for me.	0.58	
	Performing Under Pressure	1. To me, pressure situations are challenges that I welcome.	0.77	
		2. The more pressure there is during a game, the more I enjoy it	0.71	
	Goal Setting/Mental Preparation	1. On a daily or weekly basis, I set very specific goals for myself that guide what I do.	0.69	
		2. I tend to do lots of planning about how to reach my goals.	0.68	
	Concentration	1. I handle unexpected situations in my sport very well.	0.63	
		2. When I am playing sports, I can focus my attention and block out distractions	0.68	
	Free from Worry	1. While competing, I worry about making mistakes or failing to come through (**).	0.76	
		2. I put a lot of pressure on myself by worrying how I will perform (**).	0.66	
	Confidence and Achievement Motivation	1. I feel confident that I will play well.	0.65	
		2. I get the most out of my talent and skills.	0.62	
	Coachability	1. If a coach criticizes or yells at me, I correct the mistake without getting upset about it.	0.77	
		2. I improve my skills by listening carefully to advice and instruction from coaches and manager	0.57	
**Measure and Item Origin**	**Subscale**	**Items from Original Construct**	**Factor Loading**	**Author**
PERSONALITY TRAITS				
The Multidimensional Inventory of Perfectionism in Sport (Stoeber et al., 2006)	Perfectionistic Concerns	1. During training, I get completely furious if I make mistakes.	Range from 0.86 to 0.91	Items taken from the ADFS (Dunn et al., 2019)
		2. During training, I get frustrated if I do not fulfil my high expectations.		
		3. During competition, I get completely furious if I make mistakes.		
		4. During competition, I get frustrated if I do not fulfil my high expectations.		
The Sport Multidimensional Perfectionism Scale 2 (Gotwals and Dunn, 2009)	Perfectionistic Strivings	1. I feel that other athletes generally accept lower standards for themselves in sport than I do.	0.63	Items taken from the ADFS (Dunn et al., 2019)
		2. I have extremely high goals for myself in sport.	0.53	
Big Five-Inventory-10 (Gosling, Rentfrow, and Swann, 2003)	Extraversion	1. I see myself as: extraverted, enthusiastic.	0.77	Items taken from the ADFS (Dunn et al., 2019)
		2. I see myself as: reserved, quiet.		
	Agreeableness	1. I see myself as critical, quarrelsome.	0.71	
		2. I see myself as: sympathetic, warm		
	Conscientiousness	1. I see myself as: dependable, self-disciplined.	0.76	
		2. I see myself as: disorganised, careless		
	Emotional Stability	1. I see myself as: anxious, easily upset.	0.70	
		2. I see myself as: calm, emotionally stable.		
	Openness to Experiences	1. I see myself as: open to new experiences, complex.	0.62	
		2. I see myself as: conventional, uncreative.		
Life Orientation Test, (Scheier, and Carver, 1985)	Optimism	1. In uncertain times, I usually expect the best.	0.56	Items taken from the LOT (Scheier, and Carver, 1985)
		2. I always look on the bright side of things.	0.72	
		3. I’m always optimistic about my future.	0.61	
		4. I’m a believer in the idea that “every cloud has a silver lining”.	0.66	
The Brief Emotional Intelligence Scale (Davies, et al., 2010)	Appraisal of own emotions	1. I know why my emotions change.	0.77	Items taken from the BEIS-10 (Davies, et al., 2010)
		2. I easily recognise my emotions as I experience them.	0.62	
	Appraisal of others; emotions	1. I can tell how people are feeling by listening to the tone of their voice.	0.72	
		2. By looking at their facial expressions, I recognise the emotions people are experiencing.	0.65	
	Regulation of own emotions	1. I seek out activities that make me happy	0.71	
		2. I have control over my emotions	0.83	
	Regulations of others’ emotions	1. I arrange events others enjoy.	0.91	
		2. I help other people feel better when they are down	0.68	
	Utilisation of emotions	1. When I am in a positive mood, I am able to come up with new ideas.	0.65	
		2. I use good moods to help myself keep trying in the face of obstacles	0.68	
Toronto Alexithymia Scale—20 (Bagby, Parker, and Taylor, 1994)	Difficulty Identifying Feelings	1. I have feelings that I cannot quite identify	0.77	Items taken from the TAS-20 (Bagby, Parker, and Taylor, 1994)
		2. I do not know what is going on inside me	0.66	
	Difficulty Describing Feelings	1. It is difficult for me to find the right words for my feelings.	0.70	
		2. I find it hard to describe how I feel about people.	0.54	
	Externally Orientated Feelings	1. Being in touch with emotions is essential (**).	0.47	
		2. I find examination of my feelings useful in solving personal problems (**).	0.62	
**Measure and Item Origin**	**Subscale**	**Items from Original Construct**	**Factor Loading**	**Author**
PSYCHOLOGICAL FACTORS				
Athlete Burnout Measure (Raedeke, and Smith, 2001)	Emotional Exhaustion	1. I feel so tired from my training that I have trouble finding energy to do other things.	0.66	Items taken from the ABQ (Raedeke, and Smith, 2001)
		2. I feel overly tired from my [sport] participation.	0.69	
		3. I feel “wiped out” from [sport].	0.70	
		4. I feel physically worn out from [sport].	0.63	
		5. I am exhausted by the mental and physical demand of [sport].	0.70	
	Reduce Sense of Accomplishment	1. I’m accomplishing many worthwhile things in [sport].	0.67	
		2. I am not achieving much in [sport].	0.60	
		3. I am not performing up to my ability in [sport].	0.57	
		4. It seems that no matter what I do, I don’t perform as well as I should.	0.78	
		5. I feel successful at [sport].	0.66	
	Sport Devaluation	1. The effort I spend in [sport] would be better spent doing other things.	0.63	
		2. I don’t care as much about my [sport] performance as I used to.	0.50	
		3. I’m not into [sport] like I used to be	0.82	
		4. I feel less concerned about being successful in [sport] as I used to be.	0.66	
		5. I have negative feelings towards [sport].	0.65	
Perceived Stress Scale (Cohen, et al., 1983)	Global Stress and Training Stress	1. In the last week, how often have you felt that you were unable to control the important things in your life?	Range from 0.82 to 0.86	Items taken from the PSS (Cohen, et al., 1983)
		2. In the last week, how often have you felt confident about your ability to handle your personal problems? (**).		
		3. In the last week, how often have you felt that things were going your way? (**).		
		4. In the last week, how often have you felt difficulties were piling up so high that you could not overcome them?		

Key: ** = Reverse Score (i.e., 1 = 5, 2 = 4, 3 = 3, 4 = 2 and 5 = 1).
